# Interactions among Reward Sensitivity and Fast-Food Access on Healthy Eating Index Scores in Adolescents: A Cross-Sectional Study

**DOI:** 10.3390/ijerph18115744

**Published:** 2021-05-27

**Authors:** Shirlene D. Wang, Michele Nicolo, Li Yi, Genevieve F. Dunton, Tyler B. Mason

**Affiliations:** 1 Department of Preventive Medicine, University of Southern California, Los Angeles, CA 90031, USA; mnicolo@usc.edu (M.N.); dunton@usc.edu (G.F.D.); tylermas@usc.edu (T.B.M.); 2 Spatial Sciences Institute, University of Southern California, Los Angeles, CA 90089, USA; lyi632@usc.edu

**Keywords:** adolescents, cues, diet quality, fast food, food environment, reward sensitivity

## Abstract

Food cues in the environment may contribute to obesity as the consumption of unhealthy foods may reinforce reward pathways in the brain. To understand how person-level differences in reward sensitivity may be associated with diet quality, this study aimed to examine the moderating role of the availability of fast food in the environment on the relationship between reward sensitivity and diet quality in adolescents. Participants (*n* = 152; 55% female; Mage: 12.5 ± 0.93 y) completed the drive and reward subscales of the Behavioral Inhibition System (BIS)/Behavioral Activation System (BAS) Scale to assess reward sensitivity and completed two 24 h dietary recalls from which Healthy Eating Index (HEI) scores (total score and subscales of adequacy and moderation) were calculated. Fast-food environment (FFE) was operationalized as the total number of fast-food outlets within 1 km around participants’ home address. Linear regressions were used to examine the main effects and interactions between reward sensitivity and FFE in relation to HEI score. Interactions were found between FFE and BAS drive (*p* = 0.02) and BAS reward (*p* < 0.01) on HEI adequacy. There were no interaction effects on HEI moderation or HEI total scores. For individuals who had lower access exposure to fast-food outlets (−1 SD), there was a stronger positive association between higher BAS drive (t = 2.85, *p* = 0.01, 95% CI (0.35, 1.94)) and HEI adequacy scores and between higher BAS reward (t = 3.27, *p* > 0.01, 95% CI (0.72, 2.93)) and HEI adequacy scores. By examining reward sensitivity to potential food cues in residential neighborhood food environments, it is possible to understand which adolescents are more sensitive to environmental food cues and implement interventions to buffer these influences.

## 1. Introduction

The World Health Organization defines adolescence as the life period between childhood and adulthood, from ages 10 to 19 [1]. The prevalence of obesity worldwide has increased in children and adolescents (age 5–19) in recent years, from 4% in 1975 to 18% in 2016 [2]. Further, the prevalence of obesity in the U.S. has increased in adolescents in recent years, from 13.9% in 2000 to 18.5% in 2016 [3,4].

One individual difference factor that may increase obesity risk is reward sensitivity. Gray’s model of reinforcement sensitivity includes the behavioral activation system (BAS) which is sensitive to signals of reward and represents higher approach motivation [5]. A previous study in Flemish children and adolescents, ages 6 to 13 years, has suggested that reward sensitivity is associated with impulsive eating behaviors and obesity due to the rewarding nature of food [6]. Research has found in adolescents a strong relationship between heightened levels of reward sensitivity and higher levels of intake of sugar-sweetened beverages and unhealthy snacks [7]. It appears that preference is developed for foods that are fatty and sweet in individuals higher in reward sensitivity since they provide greater reinforcement value compared to less palatable food [8,9].

However, not all reward-sensitive adolescents display poor diet quality. Food availability, affordability, and desirability in adolescents’ proximal surroundings also influence dietary patterns [10,11,12]. Obesogenic food environments promote the consumption of less healthy and more unhealthy food options and are impacted by wider population factors such as food industry policies and government regulations as well as individual factors such as food availability at home or work/school and proximity to food outlets. Increased cueing and temptation for fatty and sweet foods may occur as a result of high availability and accessibility of fast food in the surrounding environment. A report by the United Nations International Children’s Emergency Fund showed that 46% of adolescents in low- and middle-income countries consume fast food at least once/week and 42% drink sugar-sweetened beverages once/day and these rates increase to 49% and 62% for high-income countries [13].

Early adolescents are typically restricted to areas in their neighborhoods accessible by walking due to their inability to drive independently, and thus are more susceptible to the food environment around their home. Consistently, some global studies have shown associations between the presence of food establishments surrounding adolescents’ homes and actual dietary patterns. A U.S. study observed a negative association between proximity to fast food and fruit or vegetable consumption and a positive association with servings of soda [14]. Australian children (10–12) with at least one fast-food outlet within 800 **m** of their home were 36% less likely to consume two or more pieces of fruit daily [15]. Further, Canadian Grade 7 and 8 students with greater proximity to convenience stores from home had lower overall diet quality scores [16]. However, a comprehensive systematic review of articles evaluating the food environment–diet relationship only found moderate overall evidence to support neighborhood food environments influencing dietary outcomes with mixed support that higher access to fast food is associated with worse diet quality and higher rates of obesity [17]. This suggests that some adolescents may be more responsive to the food environment.

While it is essential to better understand the main effects that reward sensitivity and food environment have on adolescents’ dietary behaviors, an important next step is to study the interactive role of these factors (i.e., person x environment interaction). The purpose of this study was to build upon previous work examining the association between reward sensitivity and eating behavior to cross-sectionally examine the moderating role of availability of fast-food outlets on the relationship between individual differences in reward sensitivity and dietary quality in adolescents. Based on previous research, it was hypothesized that adolescents with higher reward sensitivity would be more motivated by increased environmental cues to seek opportunities for fast-food consumption, replacing the consumption of foods more aligned with dietary recommendations, leading to poorer diet quality.

## 2. Materials and Methods

### 2.1. Methods

The Mothers’ and Their Children’s Health (MATCH) Study was a three-year longitudinal study that examined how psychosocial, behavioral, and physical factors contribute to obesity risk in dyads of mothers and children. All investigations were carried out in accordance with the Declaration of Helsinki. The study was approved by the institutional review board at the University of Southern California (HS-12-00446). Informed consent or assent was obtained from mothers and children. Participants were diverse mothers and children recruited from the greater Los Angeles metropolitan area through flyers and public school and community events. The study recruited children in 4th or 5th grade and excluded participants who had conditions that would interfere with the consent or questionnaire process. The complete protocol for the study, including full inclusion and exclusion criteria, can be found elsewhere [18].

At Wave 6 of the study, 152 early adolescents completed a paper and pencil questionnaire assessing reward sensitivity. Parental consent and child assent were obtained prior to participation. Mothers reported their child’s ethnicity, age, family income level, and sex at birth. Research staff took anthropometric measures of height and weight, and body mass index (BMI, kg/m^2^) was calculated. Height was measured by a stadiometer (PE-AIM-101) to the nearest 0.1 cm and weight was measured by a calibrated digital scale (Tanita WB-11A) to the nearest 0.1 kg. Two 24 h dietary recalls (one weekday and one weekend day) were conducted with the children over the phone by trained research staff members using the Nutritional Data System for Research (NDSR). Children were asked to recall what they ate during the most recent 24 h period (midnight to midnight) using the standardized multiple-pass interview approach. Mothers only provided assistance to clarify or were consulted for additional information when deemed necessary [19]. Healthy Eating Index (HEI) scores were calculated from the dietary data using HEI-2010 criteria [20].

### 2.2. Measures

Fast-food environment (FFE). Participants’ home addresses were geocoded at each wave and 1 km network buffers were created surrounding each location coordinate using geographic information systems (GIS) to assess for a variety of environmental context indicators. Buffering classifies what is within a given proximity based on distance outward in all directions from a point. FFE for this analysis was operationalized as the total number of the top 20 fast-food outlets in the US within 1 km around participants’ home address in 2017 as a measure of average exposure to unhealthful food cues.

Reward sensitivity. The drive and reward responsiveness subscales of the original Behavioral Inhibition System and Behavioral Activation System (BIS/BAS) scales were used [5]. Only two of the three BAS subscales were used in analysis as previous research has shown poor reliability from the fun-seeking subscale in this sample of adolescents [18]. Specifically, the drive subscale (BAS drive) (α  =  0.74) measures pursuit of desired goals, and the reward responsiveness subscale (BAS reward) (α  =  0.72) measures increased response to the prospect for reward. The subscale has been validated in previous neuroimaging and survey research [17,21]. Higher scores represent greater reward sensitivity.

Diet quality. Healthy Eating Index (HEI) is a measure of diet quality based on concordance with federal dietary guidelines from the U.S. Department of Agriculture [22]. Components that reflect the key recommendations are assigned a standard for achieving a maximum score, assessed, and scored separately per 1000 kcal and then summed to a total score out of 100. A higher total score indicates better concordance with recommendations established in the Dietary Guidelines for Americans. The HEI-2010 is made up of 12 components, including nine components associated with adequacy (encouraged food groups): total fruit, whole fruit, total vegetables, greens and beans, whole grains, dairy, total protein foods, seafood, and plant proteins and fatty acids and three components associated with moderation (dietary elements to limit): refined grains, sodium, and empty calorie consumption [20]. For the adequacy components, higher scores reflect higher intake while, for the moderation components, higher scores reflect lower intakes, which are more appropriate.

### 2.3. Data Analysis Plan

Data were processed in SPSS Version 25 for statistical analysis. All analyses were based on 152 participants. Descriptive statistics and bivariate correlations were calculated for the study measures. The SPSS PROCESS macro was used to generate linear regression models examining the main effects and interactions of BAS reward x FFE and BAS drive x FFE in relation to three diet quality outcomes: total HEI score, adequacy subscale, and moderation subscale, resulting in 6 models. Variables were centered to reduce multicollinearity and improve result interpretation before creating the interaction terms. Conditional effects generated from SPSS PROCESS were reported for significant interactions. Covariates included participants’ sex, age, BMI, ethnicity, and family income level. The significance level for statistical tests was set at 0.05.

## 3. Results

The early adolescents in the study had a mean age of 12.5 (SD = 0.93) and 55% were female. Fifty-eight percent of participants identified as Hispanic or Latino. Average BMI was 21.26 kg/m^2^ (SD= 4.90). Of the sample, 27.8% had an annual household income of <USD 35,000. Table 1 displays other participant demographics and descriptive statistics of the study variables.

Bivariate correlations showed no main effects of BAS subscales or FFE on HEI total score. BAS drive was statistically significantly correlated with FFE (r = 0.21, *p* = 0.01) and BAS reward (r = 0.41, *p* < 0.001). There was no association between BAS reward and FFE (r = −0.02, *p* = 0.82).

**Figure 1:**
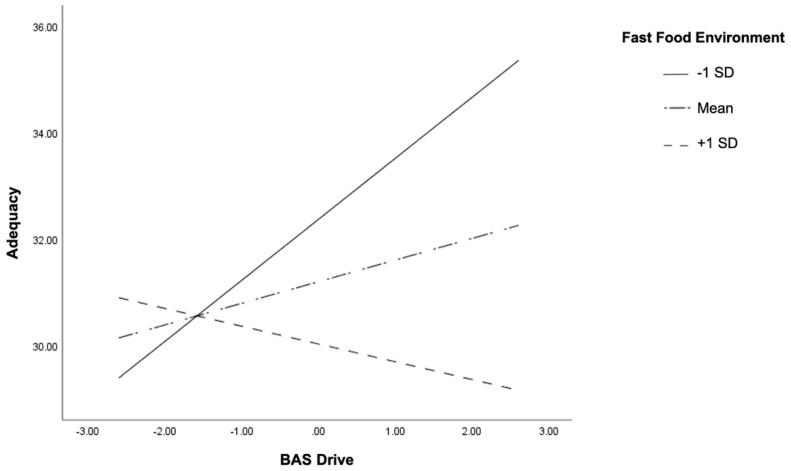
Two-way interaction of BAS drive and fast-food environment in relation to adequacy Healthy Eating Index subscores in early adolescents. High and low levels of the variables are plotted at +1 and −1 standard deviation from the mean.

**Figure 2:**
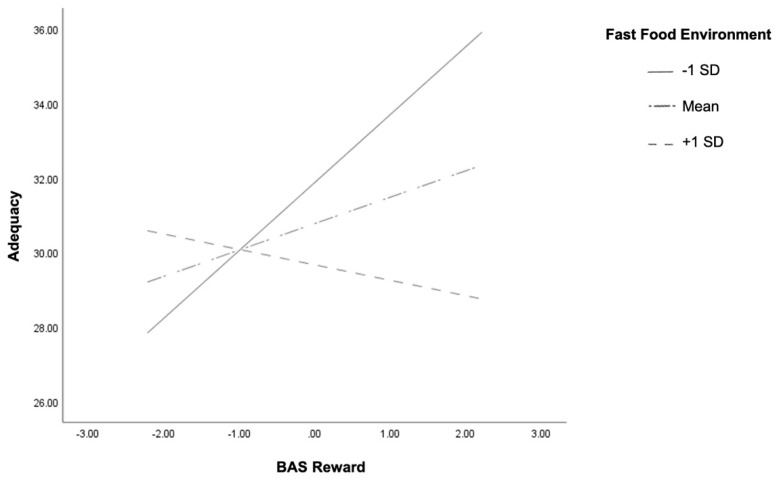
Two-way interaction of BAS reward and fast-food environment in relation to adequacy Healthy Eating Index subscores in early adolescents. High and low levels of the variables are plotted at +1 and −1 standard deviation from the mean.

Table 2 displays the multiple regression analyses for the six models. There was a main effect of FFE on HEI adequacy (*p* = 0.04) but not of BAS drive (*p* = 0.08). There was no main effect of FFE or BAS reward on HEI adequacy (*p* = 0.07 and *p* = 0.12). Interactions were found between FFE and BAS drive (*p* = 0.02) and BAS reward (*p* = 0.005) on HEI adequacy. Interactions were plotted one SD above and one SD below the mean and are displayed in Figure 1 and Figure 2, respectively.

Conditional effect analysis showed that for individuals who had lower exposure to fast-food outlets (−1 SD), there was a positive association between BAS drive and HEI adequacy scores (t = 2.85, *p* = 0.01, 95% CI (0.35, 1.94)). Higher exposure to fast-food outlets (+1 SD) showed a negative association, although it was not significant (t = −0.68, *p* = 0.50, 95% CI (−1.3, 0.63)) (Figure 1). Similar results apply to BAS reward such that individuals with lower exposure to fast food outlets (−1 SD) showed a positive association between BAS reward and HEI adequacy scores (t = 3.27, *p* > 0.01, 95% CI (0.72, 2.93)) and a non-significant association (t = −0.84, *p* = 0.40, 95% CI (−1.39, 0.56)) for adolescents living in areas with higher density of fast-food outlets (Figure 2). Therefore, higher reward sensitivity was associated with better diet adequacy in adolescents who live near less dense fast-food environments. There were no main effects or interactions of FFE or reward sensitivity on HEI moderation or total score.

We conducted post hoc power analysis for all six linear multiple regression models with G-power. Our models had eight predictors (two main effects, one interaction, five covariates) and partial R^2^ ranging from 0.12–0.17. We set alpha error prob to 0.05 and had a total sample size of 152 participants. Power (1-beta error prob) ranged from 0.91–0.99, indicating our analyses were sufficiently powered. We had power B = 0.41 to detect a small effect size (0.02), and B = 0.99 to detect a medium (0.15) and large (0.35) effect size.

## 4. Discussion

Previous research has found positive correlations between the density of fast-food outlets and poor dietary patterns, which is alarming given the recent dramatic global increase in fast food restaurants [23]. To examine whether certain adolescents may be more impacted by dense fast-food environments, the current study investigated the interactions among reward sensitivity and fast-food environment in relation to diet quality in early adolescents. We hypothesized that early adolescents with higher reward sensitivity living in more dense fast-food environments would have poorer diet quality. We found no main effects or interactions between BAS drive or reward and HEI total score or moderation, yet there was an interaction between reward sensitivity and fast-food environment in relation to adequacy. Specifically, higher reward sensitivity was associated with better diet quality among adolescents living in less dense fast-food environments.

Our finding that higher reward sensitivity was associated with greater HEI adequacy among adolescents living in less dense fast-food environments does not align with our original hypothesis. We expected that higher reward sensitivity would be associated with higher HEI moderation and lower HEI adequacy in denser fast-food environments. One reason for these unexpected findings may be that at this age, children obtain unhealthy foods though outlets other than fast-food outlets, such as vending machines, snacks after youth sports games, friends’ houses, or grocery store food purchased by parents. If true, the density of fast-food environments would contribute less to children’s need to satisfy reward craving through the consumption of fast food. Additionally, the fact that reward sensitivity was related to greater HEI adequacy in less dense fast-food environments may be due to children learning to view healthy foods as rewarding (or parents giving children healthy foods as a reward) when fast food is not as readily available. Moreover, it is possible that reward sensitivity may be adaptive in less obesogenic environments. Adolescents living in less dense FFEs may have learned to seek alternative rewards or targets for their high reward sensitivity besides food. Alternatively, due to a lack of fast food in the environment, these adolescents may eat more food at home, which typically involves food of better nutritional quality [24].

It was surprising that we only observed effects for HEI adequacy and not moderation score. It was expected that participants with higher adequacy HEI scores should be more likely to have lower moderation scores because they consume a greater amount of fruit, vegetables, and whole grains and are more likely to consume less unhealthy food. Our finding could be due to the fact that the average diet of adolescents does not conform to dietary recommendations due to the normative nature of consuming unhealthy foods [25]. While the FFE affects the association between reward sensitivity and intake of appropriate food groups, it does not seem to change the consumption of refined grains, sodium, and empty calories.

In this analysis, there was no association between the number of fast-food outlets in the home environment and HEI score. The insignificant finding may be due to our sample of younger adolescents who are less autonomous in their neighborhoods than adolescents in other studies. This finding supports a previous systematic review of papers that found higher availability and accessibility of fast food in the surrounding environment may not increase the risk of poor diet quality [17]. This also motivates our further investigation into the interaction of FFE with reward sensitivity to examine reactivity to the food environment.

No previous papers have examined the moderating role of fast-food availability on the relationship between reward sensitivity and dietary intake in adolescents. Similar to a Paquet et al. study in adults, our paper provides evidence that responsiveness to the food environment depends upon individual differences in reward sensitivity [26]. In their paper, the authors found that reward-sensitive adults living in denser fast-food environments (500 m around the home) consumed more fast food. Our results may differ due to the measure of diet used, as while Paquet et al. measured self-reports of visiting a fast-food restaurant in the past week, our study focuses on diet quality broadly. While our young adolescents had an average of 3.15 ±3.13 fast-food outlets 1 km around the home, the adults in the Paquet er al study lived in denser FFEs with 2.9 ± 2.6 fast-food outlets 500 m around the home. Moreover, there are differences between adolescents and adults such that adolescents are more reward sensitive [27] and more restricted to their home neighborhoods.

This study sheds new light on previous research by examining the interactional role of reward sensitivity and fast-food environment in relation to diet quality. The strengths of the current study include the diverse sample in regard to demographic characteristics, use of GIS to measure the fast-food environment around the early adolescents’ homes, and use of a comprehensive 24 h dietary recall to calculate the Healthy Eating Index score. However, a limitation is the cross-sectional analysis of the data which limits conclusions about the directionality of relationships. Additionally, it is possible that additional interactions would be found with a larger sample size. In the study, we only examined fast food surrounding the home environment but, since youth move between environments during the day, the fast-food environment around schools is also important to evaluate. Previous studies examining the relationship between food environment and dietary intake have found statically significant clustering of fast-food restaurants within walking distances of schools [28,29]. Additionally, participants self-reported the data used to calculate the BAS scores; however, BAS scores correlate with activation in relevant brain regions [21]. Diet quality and HEI scores of participants are subject to parent feeding and dietary patterns of the family and we cannot discern who purchased the food/drinks, drove the decision to consume the food/drinks, or whether foods/drinks were part of meals or snacks.

Increased knowledge on the associations between reward sensitivity, obesogenic food environment, and diet quality can inform the development of more effective obesity prevention interventions. As adolescents are more sensitive to reward processes than adults [27], research should continue to study this population and account for individual differences in sensitivity to reward in obesity prevention and efforts to promote healthy eating. Future research should aim to replicate these findings in a larger sample and examine if reward sensitivity is a risk factor for poor diet and obesity into adulthood using longitudinal data. Interventions may target adolescents who are high in reward sensitivity to lessen the temptation of fast food by targeting responses to food cues, improving self-regulation, or turn attention towards non-food related stimuli or transform their approach motivation toward engaging in healthy eating behaviors. Reward sensitivity is an important factor that could be associated with response to obesity and diet treatments.

## 5. Conclusions

Adolescents’ diet adequacy can be explained through an interaction of personal and environmental characteristics. In this study, we found that adolescents who were more reward sensitive had better diet quality if they lived in less dense fast-food environments. By examining reward sensitivity to potential food cues in residential neighborhood food environments, it is possible to understand which adolescents are more sensitive to environmental food cues and implement interventions to help adolescents develop the skills to resist food temptations in their neighborhoods and frequently traveled routes. Further research should longitudinally investigate associations between reward sensitivity, obesogenic food environment, and diet quality in adolescents and adults. Due to the difficult nature of policy changes, this may be more feasible than trying to lower the density of fast-food restaurants in neighborhoods or incentivizing restaurants to offer healthier food options.

## Figures and Tables

**Table 1:** Participant demographics and descriptive statistics of study variables.

	*n* = 152
Variable	Mean (SD) or N (%)
Age	12.50 (.93)
Hispanic	86 (57.72%)
Female	83 (55.33%)
BMI	21.26 (4.90)
Household Income	
<USD 35,000	32 (21.62%)
USD 35,000–USD 65,999	32 (21.62%)
USD 65,000–USD 94,999	27 (18.24%)
USD 95,000–USD 124,000	30 (20.27%)
>USD 125,000	27 (18.24%)
BAS Drive Score	10.57 (2.66)
BAS Reward Score	17.46 (2.23)
FFE	3.15 (3.13)
HEI Total Score	53.41 (0.66)
HEI Adequacy Score	30.73 (8.81)
HEI Moderation Score	22.36 (6.49)

Notes: BAS = Behavioral Activation System Scale; FFE = Fast-food environment (number of fast-food outlets within 1 km of home); HEI = Healthy Eating Index.

**Table 2:** Summary of multiple regressions of approach motivation and fast-food environment (FFE) in relation to HEI scores.

**Outcome: HEI Adequacy Score**					
Model	B	SE	t	*p*	R^2^
1					0.12
BAS Drive	0.41	0.31	1.29	0.20	
FFE	0.37	0.26	1.42	0.16	
Interaction	0.24	0.10	2.33	0.02 *	
2					0.14
BAS Reward	0.71	0.36	1.97	0.05	
FFE	0.35	0.25	1.41	0.16	
Interaction	0.36	0.12	2.90	>0.01 *	
**Outcome: HEI Moderation Score**					
Model	B	SE	t	*p*	R^2^
3					0.16
BAS Drive	0.18	0.22	0.79	0.43	
FFE	0.10	0.19	0.54	0.59	
Interaction	0.01	0.07	0.09	0.93	
4					0.17
BAS Reward	0.10	0.26	0.41	0.68	
FFE	0.09	0.18	0.53	0.60	
Interaction	0.01	0.09	0.09	0.92	
**Outcome: HEI Total Score**					
Model	B	SE	t	*p*	R^2^
5					0.14
BAS Drive	0.58	0.46	1.27	0.20	
FFE	0.47	0.38	1.24	0.22	
Interaction	0.24	0.15	1.65	0.10	
6					0.14
BAS Reward	0.60	0.53	1.15	0.25	
FFE	0.45	0.37	1.22	0.22	
Interaction	0.35	0.18	1.93	0.06	

Note: *: *p* < 0.05 significance.

## Data Availability

The data presented in this study are available on request from the corresponding author. The data are not publicly available due to the identifiable nature of the GPS data used to calculate food environment.

## References

[1] World Health Organization (2021). Adolescent Health. https://www.who.int/health-topics/adolescent-health#tab=tab_1.

[2] World Health Organization Obesity and Overweight. www.who.int/news-room/fact-sheets/detail/obesity-and-overweight.

[3] Skinner A.C., Perrin E.M., Skelton J.A. (2016). Prevalence of obesity and severe obesity in US children, 1999–2014. Obesity.

[4] Fryar C.D., Carroll M.D., Ogden C.L. Prevalence of overweight, obesity, and severe obesity among children and adolescents aged 2–19 years: United States, 1963–1965 through 2015–2016. https://stacks.cdc.gov/view/cdc/58669.

[5] Carver C.S., White T.L. (1994). Behavioral inhibition, behavioral activation, and affective responses to impending reward and punishment: The BIS/BAS Scales. J. Pers. Soc. Psychol..

[6] van den Berg L., Pieterse K., A Malik J., Luman M., Van Dijk K.W., Oosterlaan J., van de Waal H.A.D. (2011). Association between impulsivity, reward responsiveness and body mass index in children. Int. J. Obes..

[7] De Cock N., Van Lippevelde W., Goossens L., De Clercq B., Vangeel J., Lachat C., Beullens K., Huybregts L., Vervoort L., Eggermont S. (2016). Sensitivity to reward and adolescents’ unhealthy snacking and drinking behavior: The role of hedonic eating styles and availability. Int. J. Behav. Nutr. Phys. Act..

[8] Davis C., Patte K., Levitan R., Reid C., Tweed S., Curtis C. (2007). From motivation to behaviour: A model of reward sensitivity, overeating, and food preferences in the risk profile for obesity. Appetite.

[9] Epstein L.H., Leddy J.J. (2006). Food reinforcement. Appetite.

[10] Mattioni D., Loconto A.M., Brunori G. (2019). Healthy diets and the retail food environment: A sociological approach. Health Place.

[11] Egger G., Swinburn B. (1997). An ‘ecological’ approach to the obesity pandemic. BMJ.

[12] Larson N., Story M. (2009). A review of environmental influences on food choices. Ann. Behav. Med..

[13] Keeley B., Little C., Zuehlke E. (2019). The State of the World’s Children 2019: Children, Food and Nutrition--Growing Well in a Changing World.

[14] Davis B., Carpenter C. (2009). Proximity of fast-food restaurants to schools and adolescent obesity. Am. J. Public Health.

[15] Timperio A., Ball K., Roberts R., Campbell K., Andrianopoulos N., Crawford D. (2008). Children’s fruit and vegetable intake: Associations with the neighbourhood food environment. Prev. Med..

[16] He M., Tucker P., Irwin J.D., Gilliland J., Larsen K., Hess P. (2012). Obesogenic neighbourhoods: The impact of neighbourhood restaurants and convenience stores on adolescents’ food consumption behaviours. Public Health Nutr..

[17] Caspi C.E., Sorensen G., Subramanian S.V., Kawachi I. (2012). The local food environment and diet: A systematic review. Health Place.

[18] Dunton G.F., Liao Y., Dzubur E., Leventhal A.M., Huh J., Gruenewald T., Margolin G., Koprowski C., Tate E., Intille S. (2015). Investigating within-day and longitudinal effects of maternal stress on children’s physical activity, dietary intake, and body composition: Protocol for the MATCH study. Contemp. Clin. Trials.

[19] O’Connor S.G., Ke W., Dzubur E., Schembre S., Dunton G.F. (2018). Concordance and predictors of concordance of children’s dietary intake as reported via ecological momentary assessment and 24 h recall. Public Health Nutr..

[20] Guenther P.M., Casavale K.O., Reedy J., Kirkpatrick S.I., Hiza H.A., Kuczynski K.J., Kahle L.L., Krebs-Smith S.M. (2013). Update of the Healthy Eating Index: HEI-2010. J. Acad. Nutr. Diet..

[21] Beaver J.D., Lawrence A.D., van Ditzhuijzen J., Davis M.H., Woods A., Calder A.J. (2006). Individual differences in reward drive predict neural responses to images of food. J. Neurosci..

[22] Kirkpatrick S.I., Reedy J., Krebs-Smith S.M., Pannucci T.E., Subar A.F., Wilson M.M., Lerman J.L., Tooze J.A. (2018). Applications of the Healthy Eating Index for Surveillance, Epidemiology, and Intervention Research: Considerations and Caveats. J. Acad. Nutr. Diet..

[23] Zion Market Research (2017). Global Fast Food Market will reach USD 690.80 Billion in 2022: Zion Market Research. https://www.globenewswire.com/news-release/2017/03/01/929307/0/en/Global-Fast-Food-Market-will-reach-USD-690-80-Billion-in-2022-Zion-Market-Research.html.

[24] Lin B.-H., Guthrie J.F. (2012). Nutritional quality of food prepared at home and away from home, 1977–2008.

[25] Lin B., Guthrie J., Frazao E. (2001). American children’s diets not making the grade. Food Rev..

[26] Paquet C., Daniel M., Knäuper B., Gauvin L., Kestens Y., Dub L.é. (2010). Interactive effects of reward sensitivity and residential fast-food restaurant exposure on fast-food consumption. Am. J. Clin. Nutr..

[27] Hardin M.G., Ernst M. (2009). Functional Brain Imaging of Development-Related Risk and Vulnerability for Substance Use in Adolescents. J. Addict. Med..

[28] Simon P.A., Kwan D., Angelescu A., Shih M., Fielding J.E. (2008). Proximity of fast food restaurants to schools: Do neighborhood income and type of school matter?. Prev. Med..

[29] Austin S.B., Melly S.J., Sanchez B.N., Patel A., Buka S., Gortmaker S.L. (2005). Clustering of fast-food restaurants around schools: A novel application of spatial statistics to the study of food environments. Am. J. Public Health.

